# Kinetics and Mechanism of Synthesis of Carboxyl-Containing N-Vinyl-2-Pyrrolidone Telehelics for Pharmacological Use

**DOI:** 10.3390/polym13152569

**Published:** 2021-08-01

**Authors:** Andrey N. Kuskov, Anna L. Luss, Inessa A. Gritskova, Mikhail I. Shtilman, Mikhail V. Motyakin, Irina I. Levina, Anna M. Nechaeva, Oksana Yu. Sizova, Aristidis M. Tsatsakis, Yaroslav O. Mezhuev

**Affiliations:** 1 Department of Biomaterials, Mendeleev University of Chemical Technology of Russia, 125047 Moscow, Russia; al.luss@yandex.ru (A.L.L.); shtilmanm@yandex.ru (M.I.S.); anechaeva16@gmail.com (A.M.N.); oksana.sizova1991@mail.ru (O.Y.S.); 2 Department of Chemistry and Technology of Macromolecular Compounds, MIREA—Russian Technological University (RTU MIREA), 119454 Moscow, Russia; inessagritskova@gmail.com; 3 Emanuel Institute of Biochemical Physics, Russian Academy of Sciences, 119334 Moscow, Russia; motyakin@hotmail.com (M.V.M.); iilevina@inbox.ru (I.I.L.); 4 Semenov Federal Research Center for Chemical Physics, Russian Academy of Sciences, 119991 Moscow, Russia; 5 Center of Toxicology Science & Research, Division of Morphology, Medical School, Voutes Campus, University of Crete, 71003 Heraklion, Greece; aris@med.uoc.gr; 6 Department of Analytical and Forensic Medical Toxicology, Sechenov University, 119991 Moscow, Russia

**Keywords:** N-vinyl-2-pyrrolidone polymerization, polymerization mechanism, polymerization kinetics, doxorubicin

## Abstract

It was found that sulfanylethanoic and 3-sulfanylpropanoic acids are effective regulators of molecular weight with chain transfer constants of 0.441 and 0.317, respectively, and show an unexpected acceleration effect on the radical polymerization of N-vinyl-2-pyrrolidone, initiated by 2,2’-azobisisobutyronitrile. It was determined for the first time that the thiolate anions of mercapto acids form a high-temperature redox initiating system with 2,2’-azobisisobutyronitrile during the radical polymerization of N-vinyl-2-pyrrolidone in 1,4-dioxane. Considering the peculiarities of initiation, a kinetic model of the polymerization of N-vinyl-2-pyrrolidone is proposed, and it is shown that the theoretical orders of the reaction rate, with respect to the monomer, initiator, and chain transfer agent, are 1, 0.75, 0.25, and are close to their experimentally determined values. Carboxyl-containing techelics of N-vinyl-2-pyrrolidone were synthesized so that it can slow down the release of the anticancer drug, doxorubicin, from aqueous solutions, which can find its application in the pharmacological field.

## 1. Introduction

Low toxicity and a suitable rate of elimination of poly (N-vinylpyrrolidone) with a molecular weight of less than 25,000 g × mol^−1^ from the body suggest broad prospects for the use of this polymer in medicine and pharmacology [1,2]. Poly (N-vinylpyrrolidone) is traditionally used as an auxiliary component in the manufacture of tablets [3], capsules [4], hydrogels [5,6,7], solutions for oral and injection administration [8,9,10], and also as a plasma substitute [11]. The ability of poly (N-vinylpyrrolidone) to interact specifically with several pharmacologically active substances is used to regulate their solubility [12,13], as well as to create drug delivery systems [14]. For example, systems have been obtained that provide controlled release of non-steroidal anti-inflammatory drugs [15], antioxidants [16] and anticancer drugs [17,18,19]. It has been shown that the complex of poly (N-vinylpyrrolidone) with iodine is promising for the inhibition of COVID-19 [20,21,22,23]. One of the strategies for the synthesis of drug carriers based on poly (N-vinylpyrrolidone) is associated with the introduction of hydrophobic end groups as a result of the use of long-chain thiols as chain transfer agents [14,24,25,26]. This approach made it possible to ensure the selective delivery of pharmacologically active drugs into the cell nucleus [26]. In this regard, an important perspective is the introduction of terminal carboxyl groups in the poly (N-vinyl-2-pyrrolidone) chain, capable of binding amine-containing anticancer drugs due to the formation of salt ionic bonds. However, the first task on the way to the synthesis of these carriers is to study the kinetics of radical polymerization of N-vinyl-2-pyrrolidone in the presence of chain transfer agents containing both thiol and carboxyl functional groups. Although the mechanism and kinetics of radical polymerization and copolymerization of N-vinylpyrrolidone, including in the regime of pseudoliving chains RAFT and ATRP, have been the subject of discussion in a number of articles [27,28,29,30,31,32,33,34], so far there are no kinetic data obtained in the presence of thiols, and especially functionalized thiols. It should be expected that the radicals formed as a result of the monomolecular decomposition of 2,2’-azobisisobutyronitrile would interact with mercapto acids with the abstraction of a hydrogen atom; however, it turned out that the initiation mechanism is much more complicated and includes a redox reaction between the initiator and the chain transfer agent. The latter circumstance makes it possible to explain the acceleration of the radical polymerization of N-vinyl-2-pyrrolidone, initiated by 2,2’-azobisisobutyronitrile, when mercapto acids are added to the reaction system, as well as theoretically substantiate the experimentally determined kinetic orders of reagent concentrations. Therefore, this article aimed to establish the kinetics and mechanism of radical homopolymerization of N-vinylpyrrolidone in the presence of sulfanylethanoic acid, and 3-sulfanylpropanoic acid, and also address the binding aspects of the anti-cancer drug, doxorubicin.

## 2. Experimental 

### 2.1. Materials and Methods

Sulfanylethanoic acid and 3-sulfanylpropanoic acid (Sigma-Aldrich, St. Louis, Missouri, USA) were used as chain transfer agents. Polymerization of N-vinyl-2-pyrrolidone purchased from Merck and purified by vacuum distillation was carried out using 2,2’-azobisisobutyronitrile (AIBN) from Sigma-Aldrich as an initiator. The monomer conversion was determined by noting the position of the meniscus during polymerization, in an argon atmosphere, in a dilatometer placed in a thermostat. The monomer conversion was calculated using the Equation (1):

(1) $$p=\frac{d_{p}S\Delta h}{V_{M}\left(d_{p}-d_{M}\right)}$$

where: p—monomer conversion; $V_{M}$—volume of the monomer; S—cross-sectional area of the capillary; $d_{p}$, $d_{M}$—density of poly(N-vinylpyrrolidone) and monomer, respectively; Δh—vertical shrinkage of the reaction system.

The ^13^C NMR spectrum of the synthesized poly (N-vinylpyrrolidone) was recorded in D_2_O medium on a Bruker Avance 500 spectrometer (Bruker, Zurich, Switzerland) at the New Materials and Technologies Research Center at IBCP RAS. Potentiometric titration was performed using an F20-Standard pH meter, Mettler Toledo. To register the kinetics of doxorubicin release, we used a UV-vis spectrometer UNICO 2804 (“United Products & Instruments, Inc.”, Dayton, NJ, USA). Doxorubicin hydrochloride was manufactured by Sinbias Pharma (Kiev, Ukraine).

### 2.2. Study of the Polymerization (Telomerization) Kinetics of N-Vinyl-2-Pyrrolidone in the Presence of Sulfanylethanoic Acid and 3-Sulfanylpropanoic Acids

Kinetic studies were performed for AIBN-initiated block polymerization of N-vinyl-2-pyrrolidone at 343 K. The reaction was carried out at concentrations of sulfanylethanoic acid and 3-sulfanylpropanoic acid 0.09 mol × l^−1^, 0.47 mol × l^−1^, 1.4 mol × l^−1^, as well as in the absence of thiols, at an AIBN concentration of 6.3 × 10^−2^ mol × l^−1^. At a constant concentration of 0.47 mol × l^−1^ of sulfanylethanoic acid and 3-sulfanylpropanoic acid in the system, the reaction was carried out at AIBN concentrations of 3.15 × 10^−2^ mol × l^−1^, 6.30 × 10^−2^ mol × l^−1^, and 9.45 × 10^−2^ mol × l^−1^.

### 2.3. Determination of the Chain Transfer Constants for Sulfanyletanoic Acid and 3-Sulfanylpropanoic Acid

Polymerization was initiated with 1 wt% AIBN at 343 K in the presence of 0.01; 0.025; 0.05; 0.075; 0.1; 0.125; 0.15 mol of sulfanyletanoic acid (3-sulfanylpropanoic acid) per 1 mol of N-vinyl-2-pyrrolidone; a series of telechelics with different number-average degrees of polymerization was obtained. The products were isolated by vacuum filtration after precipitation into a ten-fold excess of diethyl ether and thoroughly washed with diethyl ether. The number average degrees of polymerization were determined according to Equation (2) by potentiometric titration of the end carboxyl groups of the resulting telechelics.

(2) $${\bar{X}}_{n}=\frac{m_{p}}{CVM_{NVP}}$$

where: ${\bar{X}}_{n}$—number average degree of polymerization; $m_{p}$—mass of the polymer sample; C—molarity of the KOH solution used as a titrant; V—volume of the titrant solution; $M_{NVP}$ = 111.14 g × mol^−1^.

### 2.4. Interaction of AIBN with Sulfanylethanoic and 3-Sulfanylpropane Acids

A specified amount of sulfanylethanoic acid (3-sulfanylpropanoic acid) was dissolved in a 0.2 mol × l^−1^ solution of AIBN prepared in toluene. The resulting solution was placed in an ampoule and frozen, then the ampoule was carefully sealed and transferred to a thermostat maintained at 353 K. After 20 h of keeping the solution at a temperature of 353 K, the ampoule was opened, and the volatile fractions were removed by vacuum distillation. After removing the viscous yellowish liquid by filtration, white crystals were isolated, which were washed with cold ethanol. The resulting product was weighed, and the yield was determined. The melting point of white crystals purified by recrystallization from ethanol was 441 K, and the ^1^H NMR spectrum showed a single singlet with a chemical shift of 1.54 ppm, which makes it possible to identify the obtained compound as tetramethylsuccinnitrile. ^1^H NMR spectrum was recorded in CDCl_3_ medium.

### 2.5. Immobilization of Doxorubicin on the Telomere of N-Vinyl-2-Pyrrolidone with a Terminal Thioacetic Acid Residue and the Kinetics of Release of Bound Doxorubicin

A total of 0.1 g of the synthesized telomere, N-vinyl-2-pyrrolidone, with a terminal residue of thioacetic acid, was dissolved in 5 mL of distilled water. An amount of 0.01 g of doxorubicin was dissolved in 5 mL of distilled water. The resulting solutions were mixed and dialyzed against water, followed by lyophilization. The resulting lyophilisate was dissolved in 10 mL of distilled water, and placed in a dialysis bag with a molecular weight cutoff of 500 Da (Labware supplier store 500 MWCO, Darmstadt, Germany). The kinetics of dialysis was studied at a temperature of 309 K against distilled water with a volume of 250 mL. The doxorubicin kinetics of release in the presence of poly (N-vinyl-2-pyrrolidone) 8000 g × mol^−1^ manufactured by Acros was studied by a similar method.

For comparison, we studied the kinetics of the release of doxorubicin through the dialysis membrane, (500 MWCO) from its 0.1 wt% aqueous solution under conditions similar to those described above. 

The release kinetics of doxorubicin were investigated by UV-vis spectroscopy, noting the absorbance values at 480 nm every 30 min. Kinetic measurements of dialysis rate were performed over five hours.

## 3. Results and Discussion

**Figure 1:**
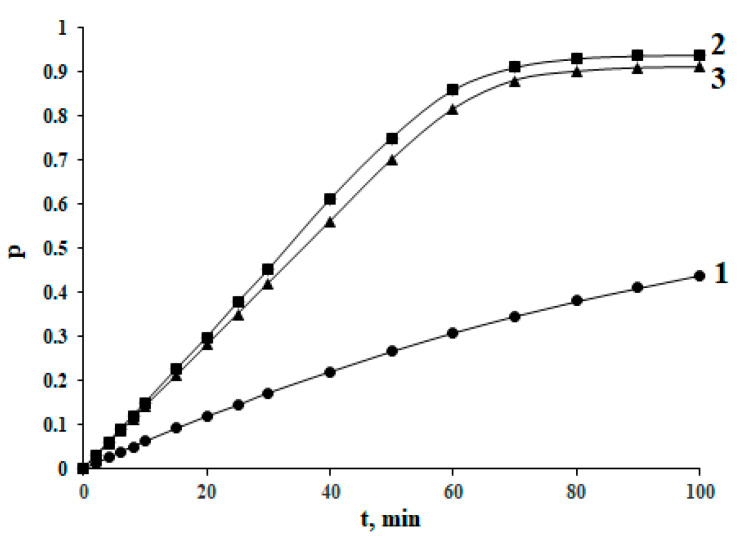
Dependences of conversion (p), on time (t), under conditions of block polymerization of N-vinyl-2-pyrrolidone at a temperature of 343 K initiated by AIBN (6.30 × 10^−2^ mol × l^−1^): 1—in the absence of chain transfer agents; 2—in the presence of 0.47 mol × l^−1^ sulfanylethanoic acid; 3—in the presence of 0.47 mol × l^−1^ 3-sulfanylpropanoic acid.

Usually, the addition of chain transfer agents does not affect the rate of free radical polymerization or lead to a slowdown. At the same time, in the presence of sulfanyletanoic acid and 3-sulfanylpropanoic acid, the rate of radical polymerization of N-vinyl-2-pyrrolidone increases significantly (Figure 1).

Acceleration of polymerization of N-vinyl-2-pyrrolidone presumably indicates the participation of mercapto acids in the act of initiation of the reaction, which limits the formation of active centers. If sulfanylethanoic acid and 3-sulfanylpropanoic acids participate in the formation of active centers, then their concentrations should be considered by the kinetic equation of polymerization. It is known that the radical polymerization of N-vinyl-2-pyrrolidone is accompanied by autoacceleration when it is carried out to high conversions of the monomer (gel effect) [27]; however, at low conversions, the order of monomer concentration is close to unity. Thus, the kinetic Equation (3) can be written:

(3) $$-\frac{dC_{M}}{dt}=kC_{I}^{i}C_{T}^{t}C_{M}=k_{eff}C_{M}$$

where: $C_{I}$, $C_{T}$, $C_{M}$—concentrations of the initiator, mercapto acids (thiols) and monomer, respectively; t—time; k, $k_{eff}=kC_{I}^{i}C_{T}^{t}$—rate constant and effective rate constant of polymerization, respectively; i, t—orders of the polymerization rate with respect to the concentrations of the initiator and mercapto acids, respectively. 

After integrating (3) for the region of low monomer conversions, when the concentrations of the initiator and mercapto acids are close to their initial values, Equation (4) was obtained.

(4) $$\ln\left(1-p\right)=-k_{eff}t$$

where: $p=\left(C_{M0}-C_{M}\right)C_{M0}^{-1}$—monomer conversion, $C_{M0}$—initial concentration of monomer.

**Figure 2:**
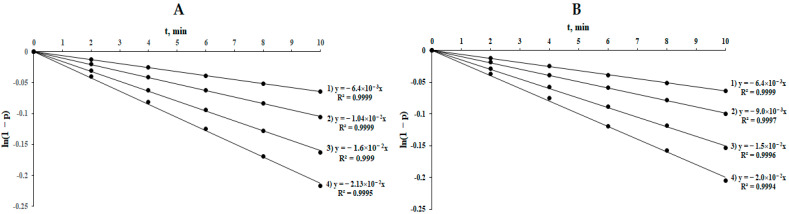
Linear dependences in coordinates ln (1 − p) vs. t in the presence of sulfanylethanoic acid (**A**) and 3-sulfanylpropanoic acid (**B**) at different initial concentrations of chain transfer agents: 1—without a chain transfer agent; 2—0.09; 3—0.47; 4—1.4 mol × l^−1^ (initial concentration of AIBN 6.30 × 10^−2^ mol × l^−1^, temperature 343 K).

**Figure 3:**
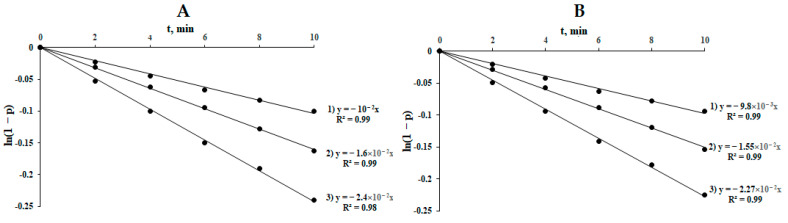
Linear dependences in coordinates ln (1 − p) vs. t in the presence of sulfanylethanoic acid (**A**) and 3-sulfanylpropanoic acid (**B**) at various initial initiator concentrations: 1—3.15 × 10^−2^; 2—6.30 × 10^−2^; 3—9.45 × 10^−2^ mol × l^−1^ (the initial concentration of chain transfer agents is 0.47 mol × l^−1^, temperature is 343 K).

The experimental kinetic data at different initial concentrations of sulfanylethanoic acid and 3-sulfanylpropanoic acids formed linear dependences in coordinates ln(1−p) vs. t (Figure 2). Straight lines in coordinates ln(1−p) vs. t were also obtained by varying the concentration of the initiator, and keeping the temperature and concentration of the introduced mercapto acids constant (Figure 3).

**Figure 4:**
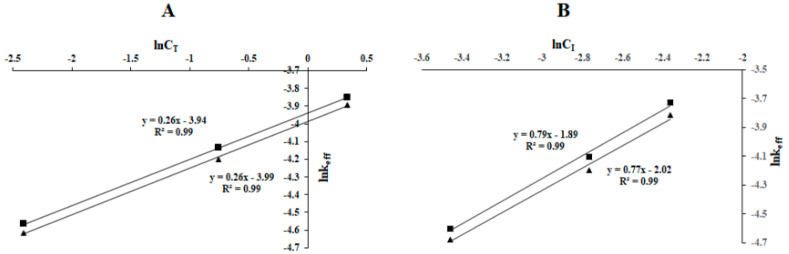
Dependence in coordinates $“lnk_{eff}\;\mathrm{vs}.\;lnC_{T}”$ (AIBN concentration is 6.30 × 10^−2^ mol × l^−1^)—**A** and $lnk_{eff}\;\mathrm{vs}.\;lnC_{I}$ (mercapto acids concentration is 0.47 mol × l^−1^)—**B** at temperature 343 K.

Effective rate constants ($k_{eff}$), determined from the slopes of the linear dependences in Figure 2 for the initial concentration of the initiator 6.30 × 10^−2^ mol × l^−1^ are 1.04 × 10^−2^; 1.60 × 10^−2^; 2.13 × 10^−2^ min^−1^ (in the presence of sulfanylethanoic acid), and 0.99 × 10^−2^; 1.50 × 10^−2^; 2.04 × 10^−2^ min^−1^ (in the presence of 3-sulfanylpropanoic acid) at mercapto acid concentrations of 0.09, 0.47, and 1.4 mol × l^−1^, respectively. In the absence of mercapto acids, $k_{eff}$ is 6.4 × 10^−3^ min^−1^. Considering (3), the obtained rate constants make it possible to determine the kinetic order of polymerization from the concentration of sulfanylethanoic and 3-sulfanylpropanoic acids, after plotting the dependences $lnk_{eff}\;\mathrm{vs}.\;lnC_{T}$ in accordance with Equation (5) (Figure 4A).

(5) $$lnk_{eff}=\ln\left(kC_{I}^{i}\right)+tlnC_{T}$$

Effective rate constants of reaction ($k_{eff}$), calculated from the tangent of the slope of the straight lines in Figure 3, are 10^−2^; 1.60 × 10^−2^; 2.40 × 10^−2^ min^−1^ (in the presence of sulfanylethanoic acid), and 0.93 × 10^−2^; 1.50 × 10^−2^; 2.20 × 10^−2^ min^−1^ (in the presence of 3-sulfanylpropanoic acid) at initiator concentrations of 3.15 × 10^−2^, 6.30 × 10^−2^ and 9.45 × 10^−2^ mol×l^−1^, respectively, if the initial concentration of mercapto acids is 0.47 mol × l^−1^. Thus, the order of radical polymerization of N-vinyl-2-pyrrolidone based on the initiator concentration can be determined from the linear dependence $lnk_{eff}\;\mathrm{vs}.\;lnC_{I}$ in accordance with Equation (6) (Figure 4B).

(6) $$lnk_{eff}=\ln\left(kC_{T}^{t}\right)+ilnC_{I}$$

The results indicate that the order of concentration of AIBN is 0.77 and 0.79, when sulfanylethanoic acid and 3-sulfanylpropanoic acid are used as chain transfer agents, respectively. The kinetic order, in terms of the concentration of mercapto acids, in both cases is 0.26. The order in terms of the concentration of N-vinyl-2-pyrrolidone is close to unity with its low conversion, which is confirmed by the linearity of the dependences shown in Figure 2 and Figure 3. Thus, the replacement of sulfanylethanoic acid by 3-sulfanylpropanoic acid, as expected, does not cause a change in the reaction orders for all components. 

The kinetic order, in terms of the AIBN concentration in radical polymerization, is usually close to 0.5, which is a consequence of bimolecular chain termination. The chain termination mechanism, which affects the order of the reaction rate with respect to the initiator in the radical polymerization of N-vinylpyrrolidone, remains a subject of discussion [35]. The possibility of both traditional bimolecular chain termination, [36] and first-order chain termination, in terms of the concentration of active centers, which are often associated with the participation of impurities in chain termination [27,35,37], has been shown. However, the development of the gel effect at high conversions of N-vinyl-2-pyrrolidone, noted in [27], rather indicates the realization of bimolecular chain termination.

**Scheme 1:**
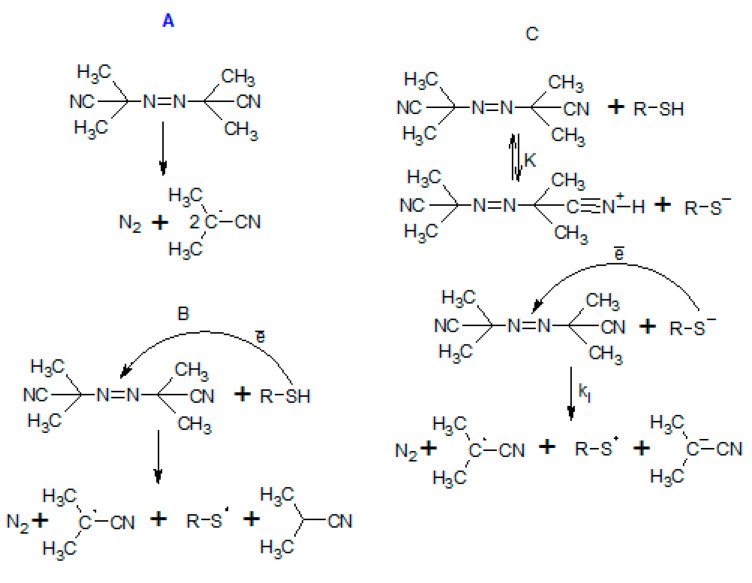
Possible mechanisms of formation of radicals initiating polymerization: (**A**)—due to thermolysis of AIBN; (**B**)—due to the redox reaction of AIBN and mercapto acids; (**C**)—due to the redox reaction of AIBN and thiolate anions of mercapto acids (dianions of mercapto acids).

The data obtained also indicate the participation of mercapto acids and AIBN in the formation of active centers at the initiation stage (Figure 4). Therefore, it is necessary to compare the experimental orders of the polymerization rate of N-vinyl-2-pyrrolidone, based on the concentrations of AIBN and mercapto acids, with the possible mechanisms of the formation of radicals at the initiation stage. As alternatives, consider the monomolecular thermolysis of AIBN (Scheme 1A), the redox reaction between AIBN and mercapto acids (Scheme 1B), and the oxidation of thiolate anions of mercapto acids under the action of AIBN (Scheme 1C).

Assuming that there is bimolecular chain termination, we can write the kinetic Equation (7) [38].

(7) $$R=-\frac{dC_{M}}{dt}=\frac{k_{p}}{k_{t}^{0.5}}R_{I}^{0.5}C_{M}$$

where: $k_{p}$—chain growth rate constant; $k_{t}$—breakage chain rate constant; $R_{I}$—initiation rate of radical polymerization; R—rate of radical polymerization.

If thermolysis of the initiator is realized (Scheme 1A), then $R_{I}\;\sim \;C_{I}$ and, therefore, $R\;\sim \;C_{I}^{0.5}C_{M}$. In this case, the reaction rate does not depend on the concentration of mercapto acids, and the expected kinetic order with respect to the initiator concentration is 0.5, which contradicts the experimental data. The assumption of a redox reaction between thiols and AIBN (Scheme 1B) allows us to conclude that $R_{I}\;\sim \;C_{I}C_{T}$ and $R\;\sim \;C_{I}^{0.5}C_{T}^{0.5}C_{M}$. In this case, the polymerization rate depends on the concentration of mercapto acids; however, the orders in the concentrations of the initiator and chain transfer agents do not correspond to their experimentally determined values. Assuming that the act of one-electron oxidation is preceded by the deprotonation of the thiol group under the action of AIBN, it is possible to write Equation (8) (Scheme 1C).

(8) $$K=\frac{C_{AIBNH^{+}}C_{RS^{-}}}{C_{I}C_{T}}=\frac{C_{RS^{-}}^{2}}{C_{I}C_{T}}$$

where: $C_{AIBNH^{+}}=C_{RS^{-}}$—equilibrium concentrations of protonated AIBN and thiolate ion, respectively; *K*—constant of acid-base equilibrium. Thus, the equilibrium concentration of thiolate anion and the rate of initiation can be expressed by Equations (9) and (10), respectively.

(9) $$C_{RS^{-}}={\left(KC_{I}C_{T}\right)}^{0.5}$$

(10) $$R_{I}=k_{I}K^{0.5}C_{T}{}^{0.5}C_{I}{}^{1.5}$$

where: $k_{I}$—initiation rate constant.

Substitution of Equation (10) into Equation (7) leads to kinetic Equation (11) for the total polymerization rate.

(11) $$R=\frac{k_{p}}{k_{t}^{0.5}}k_{I}^{0.5}K^{0.25}C_{T}{}^{0.25}C_{I}{}^{0.75}C_{M}$$

Equation (11) corresponds to the experimentally determined orders of the polymerization rate with respect to the concentrations of mercapto acids and initiator. Thus, the kinetic data indicate the one-electron oxidation of thiolate anions of mercapto acids, under the action of AIBN, which is the cause of the formation of radicals initiating polymerization. Thus, we assume that the low equilibrium concentration of thiolate anions of mercapto acids (dianions of mercapto acids) is compensated by their high reducing activity [39]. In this case, the dissociation of the carboxyl group (the formation of monoanions of mercapto acids), which has a higher acidity than the thiol group, obviously cannot significantly affect the reducing properties of mercapto acids. 1,4-dioxane and AIBN can act as bases in the reaction system; however, only protonation of AIBN, under the action of mercapto acid monoanions, leads to the formation of particles active in single electron transfer.

**Figure 5:**
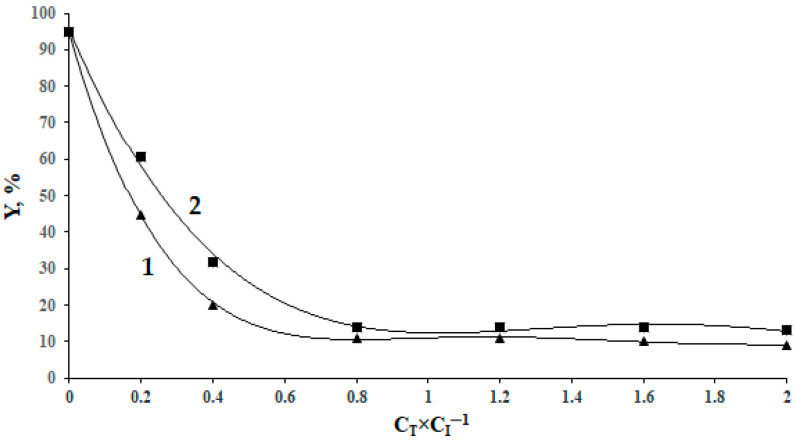
Dependence of the yield of tetramethylsuccinonitrile on the ratio of the concentrations of sulfanylethanoic acid—1 (3-sulfanylpropanoic acid—2) and AIBN after heating for 20 h at a temperature of 353 K.

If we assume that the radicals formed by the reaction shown in Scheme 1C statistically recombine, then the theoretical yield of tetramethyl succinonitrile at an equimolar ratio of reagents is 12.5%, based on the initial AIBN. When studying the decomposition of AIBN in the presence of sulfanylethanoic and 3-sulfanylpropanoic acids, without N-vinyl-2-pyrrolidone at an equimolar ratio of reagents, the yield of tetramethylsuccinonitrile in both cases was about 13% (Figure 5), which also agrees with the initiation of polymerization according to Scheme 1C. 

**Figure 6:**
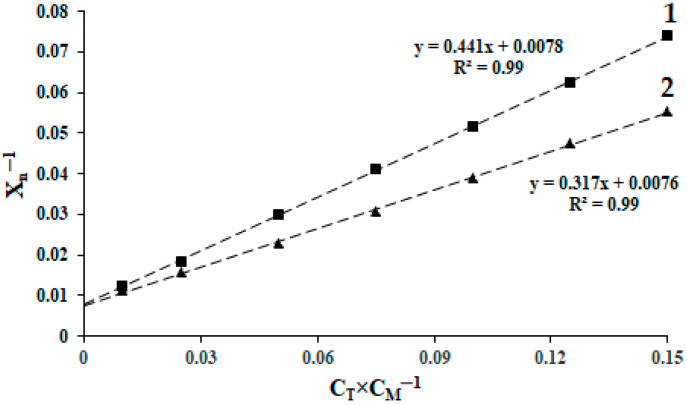
Linear dependences ${\bar{X}}_{n}^{-1}\;\mathrm{vs}.\;C_{T}C_{M}^{-1}$, for radical polymerization of N-vinyl-2-pyrrolidone at 343K in the presence of sulfanyletanoic acid—1 and 3-sulfanylpropanoic acid—2.

Sulfanylethanoic acid and 3-sulfanylpropanoic acids are active chain transfer agents, which is confirmed by a significant decrease in the number average degree of polymerization of poly (N-vinyl-2-pyrrolidone), even at a low concentration of these mercapto acids. The construction of linear dependences, ${\bar{X}}_{n}^{-1}\;\mathrm{vs}.\;C_{T}C_{M}^{-1}$ (Figure 6) in accordance with the Mayo Equation (12) allows us to determine the chain transfer constants to sulfanylethanoic acid and 3-sulfanylpropanoic acids.

(12) $${\bar{X}}_{n}^{-1}={\bar{X}}_{n0}^{-1}+\lambda_{T}C_{T}C_{M}^{-1}$$

where: $\lambda_{T}$—relative constant of chain transfer to mercapto acids; ${\bar{X}}_{n0}$—number average degree of polymerization of the polymer formed in the absence of mercapto acids.

Experimental data indicate a higher activity of sulfanylethanoic acid ($\lambda_{T}=0.441$), compared to 3-sulfanylpropanoic acid ($\lambda_{T}=0.317$). The calculated $\lambda_{T}$ values are consistent with a greater stabilization by the inductive effect of the carboxyl group of the radical formed from sulfanylethanoic acid, as compared to its homologue formed from 3-sulfanylpropanoic acid. Probably for this reason, the rate of polymerization of N-vinyl-2-pyrrolidone in the presence of sulfanylethanoic acid is somewhat higher than with the addition of 3-sulfanylpropanoic acid.

**Figure 7:**
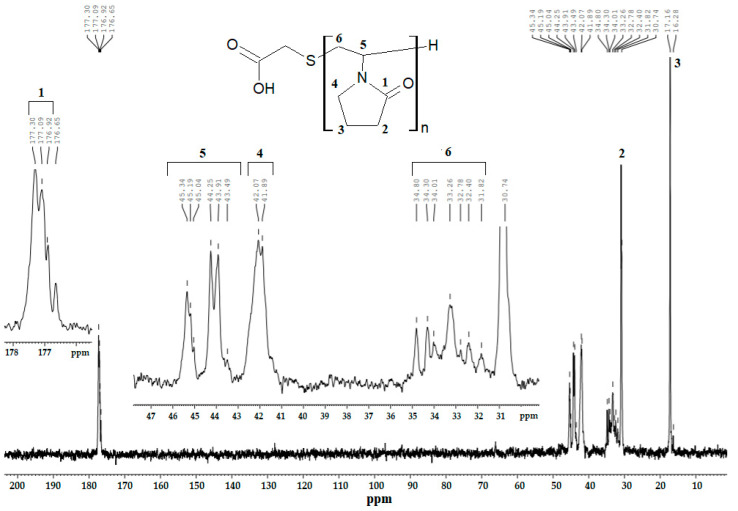
^13^C NMR spectrum (in D_2_O solution) of the telomer of N-vinyl-2-pyrrolidone obtained in the presence of sulfanylethanoic acid.

The high value of $\lambda_{T}$ make it possible to achieve selectivity with respect to end groups for telomers of N-vinyl-2-pyrrolidone formed in the presence of mercapto acids. The ^13^C NMR spectrum of the telomer of N-vinyl-2-pyrrolidone has a signal of 176.65 ppm., which corresponds to the terminal carboxylate and is clearly distinguishable against the background of the signal, in the range of chemical shifts 176.92–177.30 ppm, which belongs to the amide group of N-vinyl-2-pyrrolidone residues of various configurations. In addition, the chemical shift region is 115–125 ppm. does not contain signals characteristic of carbon atoms of the nitrile group, which indicates an insignificant contribution of the processes of interaction of the monomer and radicals formed directly during the decomposition of AIBN to the formation of polymer chains. The position and structure of the remaining signals in the ^13^C NMR spectrum of the synthesized telomere of N-vinyl-2-pyrrolidone correspond to their assignment (Figure 7), considering the various configurations of chain links described earlier in the article [40].

**Scheme 2:**
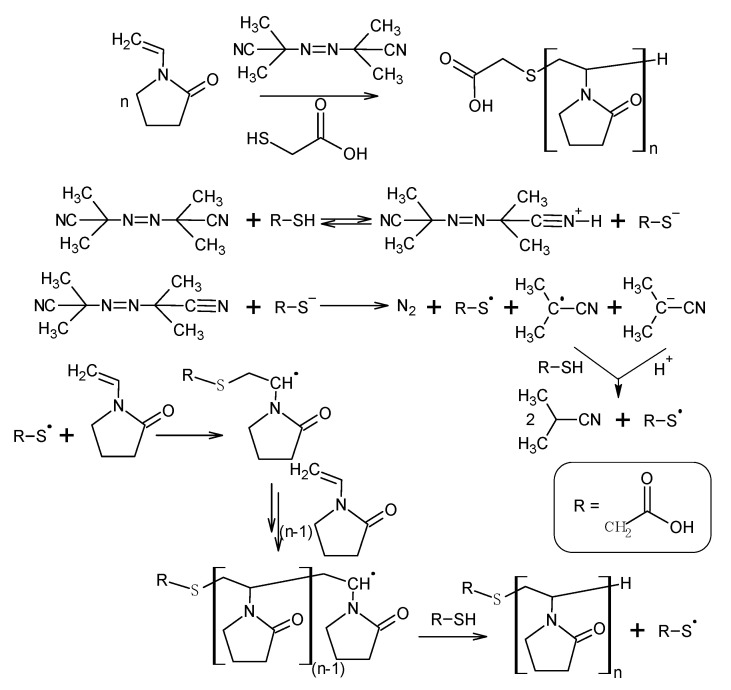
Mechanism of radical telomerization of N-vinyl-2-pyrrolidone in the presence of sulfanylethanoic acid.

The mechanism of radical telomerization of N-vinyl-2-pyrrolidone in the presence of sulfanylethanoic acid is shown in Scheme 2.

**Figure 8:**
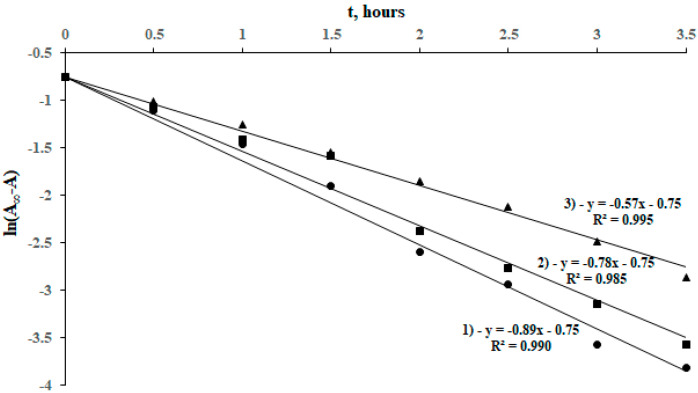
Linear dependence $\ln\left(A_{\infty }-A\right)$ vs. t, obtained for the process of release of doxorubicin at a temperature of 309 K through the dialysis membrane (500 MWCO): 1—from an aqueous solution; 2—from an aqueous solution of poly(N-vinyl-2-pyrrolidone); 3—from an aqueous solution of telechelic N-vinyl-2-pyrrolidone with terminal residues of thioacetic acid.

Telechelic of N-vinyl-2-pyrrolidone synthesized in the presence of sulfanylethanoic acid is capable of non-covalent binding of doxorubicin, which has a broad spectrum of anticancer activity [41,42,43]. Investigation of the rate of release of doxorubicin from a solution in distilled water in the presence of telechelic N-vinyl-2-pyrrolidone with terminal residues of thioacetic acid, and in its absence, showed satisfactory compliance of the experimental dependences with the kinetic Equation of the pseudo-first order (13) (Figure 8).

(13) $$\ln\left(A_{\infty }-A\right)=-k_{r}t+C$$

where: A, $A_{\infty }$—current value and maximum value of the optical density of the solution at a wavelength of 480 nm; $k_{r}$—the rate constant of doxorubicin release; *C*—the constant of integration.

The pseudo first order Equation (13) is also valid in the case of the release of doxorubicin in the presence of poly (N-vinyl-2-pyrrolidone), which does not contain a terminal carboxyl group with a molecular weight close to the molecular weight of carboxyl-containing telechelic (Figure 8).

The rate constant of the release of pure doxorubicin from an aqueous solution through the dialysis membrane is 0.89 h^−1^. The introduction of telechelic N-vinyl-2-pyrrolidone with terminal residues of thioacetic acid leads to a decrease in the rate constant of the release of doxorubicin to 0.57 h^−1^, while in both cases the same limiting value of the optical density of solutions is achieved. In the presence of poly (N-vinyl-2-pyrrolidone), which does not contain a terminal carboxyl group, the doxorubicin release rate constant is 0.78 h^−1^ (Figure 8).

**Scheme 3:**
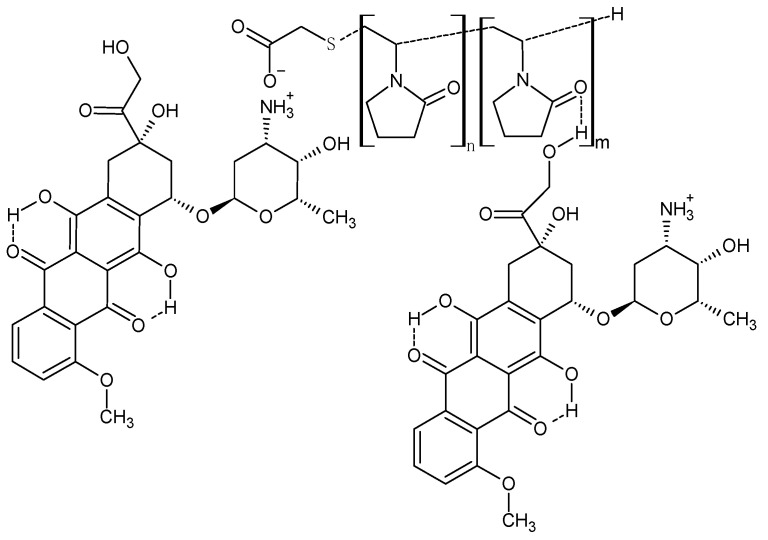
Supposed interactions between protonated doxorubicin and carboxyl-containing telechelik N-vinyl-2-pyrrolidone.

It should be expected that the dissociation constant of the terminal carboxyl group of telechelic N-vinyl-2-pyrrolidone is close to the dissociation constant of sulfanylethanoic acid, and is about 4 × 10^−4^, while the acidity constant of the alkylammonium fragment of the doxorubicin molecule is 3.47 × 10^−9^. At a concentration of 1.12 × 10^−3^ mol × l^−1^ of the terminal carboxyl groups of telechelic N-vinyl-2-pyrrolidone and a concentration of 1.84 × 10^−3^ mol × l^−1^ of aminoalkyl fragments of doxorubicin, the former are almost completely ionized, while the degree of dissociation of the latter is only 0.18%. Therefore, a significant contribution to the delay in the release of doxorubicin is made by electrostatic interactions between the ionized carboxyl group of telechelic N-vinyl-2-pyrrolidone and the salt form of doxorubicin. However, poly (N-vinyl-2-pyrrolidone), lacking a terminal carboxyl group, also slows down the release of doxorubicin, but to a lesser extent than a carboxyl-containing polymer. Therefore, it can be assumed that the formation of hydrogen bonds somewhat contributes to the delay in the release of doxorubicin in the presence of poly (N-vinyl-2-pyrrolidone) (Scheme 3).

Thus, in the presence of the carboxyl-containing telechelic N-vinyl-2-pyrrolidone, the rate of release of doxorubicin during dialysis is significantly reduced, which makes it possible to count on an increase in the circulation time of the drug in the vascular bed after injection. A decrease in the rate of absorption of doxorubicin may contribute to a decrease in its cardiotoxic effect, which is the main side effect of therapy [44,45,46,47].

## 4. Conclusions

It was found that sulfanylethanoic acid and 3-sulfanylpropanoic acids are not only effective regulators of molecular weight with relative chain transfer constants of 0.441 and 0.317, respectively, but also accelerate the radical polymerization of N-vinyl-2-pyrrolidone. The acceleration of the radical polymerization of N-vinyl-2-pyrrolidone in the presence of sulfanylethanoic and 3-sulfanylpropanoic acids is explained by their participation in the initiation act. It is shown that the experimental values of the polymerization rate orders with respect to N-vinyl-2-pyrrolidone, initiator, and mercapto acids are 1, 0.77–0.79, and 0.26, respectively. The experimental values of the orders are close to theoretical, if we assume that the initiation mechanism includes one-electron oxidation of thiolate anions of mercapto acids under the action of 2,2’-azobisisobutyronitrile. Thus, it has been shown for the first time that mercapto acids and 2,2’-azobisisobutyronitrile form a high-temperature redox initiating system, causing radical polymerization of N-vinyl-2-pyrrolidone.

The use of mercapto acids as chain transfer agents in the radical polymerization of N-vinyl-2-pyrrolidone provides selective introduction of terminal carboxyl groups. It has been shown that the addition of sulfanylethanoic acid makes it possible to obtain a carboxyl-containing telechelic of N-vinyl-2-pyrrolidone capable of slowing down the release of doxorubicin from aqueous solutions.

## Data Availability

The data presented in this study are available on request from the corresponding author.
